# Medical Classes in St. Louis

**Published:** 1858-01

**Authors:** 


*Medical Classes in St. Louis.*—The number of students at present in attendance in our Medical Colleges is considerably greater than at a corresponding period last year, and the probabilities are that we shall have larger classes this winter. The "hard times," which are bearing so heavily on the commercial interests of the land, seem not to have reached the farmers in the country, and will in all probability not affect the Medical Schools injuriously. Indeed, we believe it will hold true, as a general rule, that when a check is given to commerce and speculation, the effect will be to increase, rather than to diminish, the number of those seeking admission to the learned professions, as by these reverses men are brought to realize the fact that the slow way of making a living is, after all, the better way.

The public Introductory Lecture before the class in the St. Louis Medical College was delivered on Monday evening, November 2d, by Prof. Charles W. Stevens; and that before the Missouri Medical College on Tuesday evening, November 3d, by Prof. S. G. Armor.—*St. Louis Med. and Surg. Journal.*

